# Ouabain Does Not Induce Selective Spiral Ganglion Cell Degeneration in Guinea Pigs

**DOI:** 10.1155/2018/1568414

**Published:** 2018-07-31

**Authors:** Timo Schomann, Dyan Ramekers, John C. M. J. de Groot, Carola H. van der Ploeg, Ferry G. J. Hendriksen, Stefan Böhringer, Sjaak F. L. Klis, Johan H. M. Frijns, Margriet A. Huisman

**Affiliations:** ^1^Department of Otorhinolaryngology and Head & Neck Surgery, Leiden University Medical Center, 2300 RC Leiden, Netherlands; ^2^Percuros B.V., 7522 NB Enschede, Netherlands; ^3^Department of Otorhinolaryngology and Head & Neck Surgery, University Medical Center Utrecht, 3508 GA Utrecht, Netherlands; ^4^Brain Center Rudolf Magnus, University Medical Center Utrecht, 3508 GA Utrecht, Netherlands; ^5^Department of Medical Statistics and Bioinformatics, Leiden University Medical Center, 2300 RC Leiden, Netherlands

## Abstract

Round window membrane (RWM) application of ouabain is known to selectively destroy type I spiral ganglion cells (SGCs) in cochleas of several rodent species, while leaving hair cells intact. This protocol has been used in rats and Mongolian gerbils, but observations in the guinea pig are conflicting. This is why we reinvestigated the effect of ouabain on the guinea pig cochlea. Ouabain solutions of different concentrations were placed, in a piece of gelfoam, upon the RWM of the right cochleas. Auditory function was assessed using acoustically evoked auditory brainstem responses (aABR). Finally, cochleas were fixed and processed for histological examination. Due to variability within treatment groups, histological data was pooled and three categories based upon general histological observations were defined: cochleas without outer hair cell (OHC) and SGC loss (Category 1), cochleas with OHC loss only (Category 2), and cochleas with OHC and SGC loss (Category 3). Animals treated with 1 mM or 10 mM ouabain showed shifts in hearing thresholds, corresponding with varying histological changes in their cochleas. Most cochleas exhibited complete outer hair cell loss in the basal and middle turns, while some had no changes, together with either moderate or near-complete loss of SGCs. Neither loss of inner hair cells nor histological changes of the stria vascularis were observed in any of the animals. Cochleas in Category 1 had normal aABRs and morphology. On average, in Category 2 OHC loss was 46.0±5.7%, SGC loss was below threshold, ABR threshold shift was 44.9±2.7 dB, and ABR wave II amplitude was decreased by 17.1±3.8 dB. In Category 3 OHC loss was 68.3±6.9%, SGC loss was 49.4±4.3%, ABR threshold shift was 39.0±2.4 dB, and ABR amplitude was decreased by 15.8±1.6 dB. Our results show that ouabain does not solely destroy type I SGCs in the guinea pig cochlea.

## 1. Introduction

In the last decades cochlear implant (CI) technology has progressed considerably, resulting in a variety of improvements such as advanced electrode and speech-processor strategies associated with high performance levels. However, crucial for CI efficacy is the preservation of a critical number of auditory neurons [1, 2]. In this perspective, CI users could benefit from stem-cell-based therapy, especially since stem cells may introduce a healthy, additive population of neurons and glial cells that is necessary for interaction with and repair of the damaged auditory nerve [3, 4]. Several studies have already investigated the use of stem cells in auditory neuron regeneration, showing that various types of stem cells have the capacity to migrate either centrally or peripherally to functionally appropriate locations in the inner ear [3–7].

In future transplantation experiments, we intend to study the potential of hair-follicle-bulge-derived stem cells to repair the damaged auditory nerve in a guinea pig model. Such a study calls for partial denervation of the auditory nerve without inflicting damage to the organ of Corti, in particular, loss of inner hair cells and supporting cells. The approach of our choice is round window membrane application of ouabain, a selective and potent Na^+^/K^+^-ATPase inhibitor, which has been reported to result in selective degeneration and subsequent loss of type I spiral ganglion cells (SGCs) without affecting the type II SGCs and the hair cells in the organ of Corti ([8, 9]; for a review, see [10]). The advantage of a protocol that selectively destroys (type I) SGCs would be that the remaining hair cells and supporting cells may continue to produce chemotactic growth factors, supporting transplanted stem cells, and directing the peripheral projections of newly formed neurons to the hair cells in the organ of Corti [11–13].

The denervatory effect of ouabain was originally observed in the cochlea of Mongolian gerbils by Schmiedt and coworkers [8, 9] and corroborated by other authors [14–16]. It was subsequently demonstrated to occur as well in the cochlea of other rodent species, such as mouse [17–22] and rat [23–25], mostly in a dose-dependent way. It has also been reported that ouabain destroys SGCs in the cochlea of the cat without causing any hair cell loss [26].

Ouabain has been used in a number of stem cell transplantation studies as an agent to induce selective loss of type I SGCs in the cochlea prior to transplantation of stem cells, mostly in Mongolian gerbils [6, 12, 27–29], but also in mice [30] and rats [31] as well as in guinea pigs [11].

Only two reports have described the morphological effect of local application of ouabain upon the SGCs in the guinea pig cochlea. Hamada and Kimura [32] investigated the effect of ouabain after round window application upon cochlear histology and observed that ouabain causes, in a dose-dependent way, shrinkage (and loss) of type I SGCs, loss of primarily outer hair cells (OHCs), degeneration of the nerve endings at the base of the inner hair cells (IHCs), and, in the most severely affected cases, edema in the stria vascularis. This is in stark contrast to the paper by Cho et al. [11] who found that the effect of round window membrane application of ouabain in the guinea pig cochlea is similar to that observed in other rodents, i.e., that it selectively destroys the type I SGCs without affecting the number of hair cells and the morphological appearance of the stria vascularis.

These conflicting papers led us to reinvestigate the effects of ouabain application via the round window membrane of the guinea pig cochlea and to study the validity of this protocol in the guinea pig.

## 2. Material and Methods

### 2.1. Animals and Experimental Design

Twenty healthy albino guinea pigs (*Cavia porcellus*; strain: Dunkin Hartley; weighing 250-350 g; 4 males and 16 females) were obtained from Envigo RMS B.V. (Horst, the Netherlands). The animals were housed in the Animal Care Facility of Utrecht University (Netherlands) under standard housing conditions (group cages with enriched environment; diurnal light cycle (12 h light, 12 h dark); temperature 21°C; relative humidity 60%) and had free access to food and water.

The animals underwent surgery during which a piece of gelfoam soaked in ouabain solutions of different concentrations (Groups I-IV) was placed upon the round window membrane of the right cochleas. Group I (n=2) was treated with a 10 mM solution; Group II (n=8) with a 1 mM solution; Group III (n=4) with a 0.1 mM solution; and Group IV (n=4) with a 0.01 mM solution (Table 1). A control group (Group V; n=2) was treated with a piece of gelfoam soaked in sterile phosphate-buffered saline (PBS). Due to the unexpected variability of the effects with 1 mM ouabain we decided to investigate also the effect of 10 mM ouabain, which was our stock solution, since we expected the electrophysiological and histological effect of ouabain to be unambiguous. However, the number of animals in our animal experimental permit was limited due to ethical issues and redesign during the course of the experiments. The left cochleas were not treated and served as normal-hearing controls. Auditory brainstem responses were recorded to determine the baseline (pretreatment) hearing thresholds (Day 0) and posttreatment hearing thresholds (Days 2, 4, and 7). Animals were euthanized at either Day 4 (Group I) or Day 7 (Groups II-V).

The surgical and experimental procedures used in this study were approved by the Animal Experiments Committees of both Leiden University Medical Center (DEC permit 13224) and the University Medical Center Utrecht (DEC 2015.I.03.004). Animal care and handling were in accordance with the guidelines and regulations as stipulated by the Dutch Experiments on Animals Act (WoD) and the European Directive on the Protection of Animals Used for Scientific Purposes (2010/63/EU).

### 2.2. Ouabain Preparation

Ouabain octahydrate (Sigma Aldrich, St. Louis, MO, USA) was dissolved in sterile PBS at a concentration of 10 mM. This stock solution was aliquoted and stored at 4°C, shielded from the light. Prior to use, aliquots were diluted in PBS to prepare working solutions with a final concentration of either 10 mM, 1 mM, 0.1 mM, or 0.01 mM. Immediately before surgery, small pieces (1 mm^3^) of gelfoam (Willospon® Special; Will-Pharma, Zwanenburg, the Netherlands) were soaked in 10 *μ*l of working solution [33]. The gelfoam carrier was used once the fluid was completely absorbed.

### 2.3. Deafening Procedure

Animals were anesthetized by combined intramuscular (i.m.) injection of (S)-ketamine (Narketan®; Vétoquinol, France; 40 mg/kg) and dexmedetomidine (Dexdomitor®; Orion Pharma, Finland; 0.25 mg/kg).

Both preemptive analgesia and antibiotic prophylaxis were given preoperatively and consisted of subcutaneous (s.c.) injections of the nonsteroidal anti-inflammatory drug carprofen (Rimadyl®; Zoetis B.V., Capelle a/d IJssel, the Netherlands; 5 mg/kg) and the nonototoxic antibiotic enrofloxacin (Baytril® 5%; Bayer AG, Leverkusen, Germany; 5 mg/kg), respectively. No adverse interactions between these drugs and ouabain have been reported in the pharmaceutical databases (cf., https://www.drugbank.ca).

**Figure 1:**
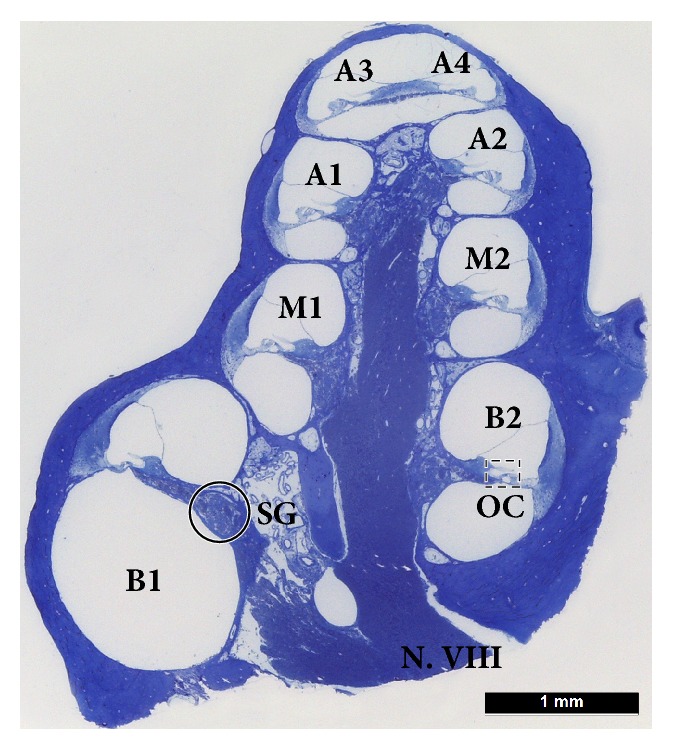
Histology of the guinea pig cochlea. The midmodiolar section of a guinea pig cochlea shows 8 different locations (basal = B1, B2; middle = M1, M2; apical = A1, A2, A3, and A4) along the cochlear spiral at a half-turn spacing (not showing the helicotrema). The number of SGCs in the spiral ganglion (SG) were counted and the number of hair cells (IHCs and OHCs) in the organ of Corti (OC) was determined for each location, except A4.

Prior to surgery, the site of incision was infiltrated subcutaneously with 0.3 ml of a local anesthetic mixture containing 2% xylocaine and adrenaline 1:200,000 (AstraZeneca, London, UK). A retroauricular incision was made in order to expose and open the auditory bulla. A hole was made ventrolaterally in the posterior portion of the right auditory bulla (Figure 1) using a #15 surgical blade under the operating microscope to reveal the round window. A piece of gelfoam soaked with ouabain or PBS was placed into the round window niche [33]. The hole in the bulla was fully closed with self-curing glass ionomer restorative dental cement (GC Fuji PLUS® II; GC Corporation, Tokyo, Japan) followed by suturing the wound with either Safil® 5/0 (B. Braun Melsungen AG, Melsung, Germany) or Vicryl® 5/0 (Ethicon, Somerville, NJ, USA) absorbable surgical suture. Immediately after surgery, atipamezole (Antisedan®; Orion Pharma; 1 mg/kg) was injected subcutaneously to abolish the clinical effects of dexmedetomidine. One day after surgery, a second s.c. injection of carprofen (5 mg/kg) was given.

### 2.4. Auditory Brainstem Responses

Auditory function of the right and left ears was separately assessed using acoustically evoked auditory brainstem responses (aABRs) before surgery (Day 0) and at 2, 4, and 7 days after gelfoam placement.

Prior to auditory testing on Days 2, 4 and 7, anesthesia was induced by combined i.m. injection of (S)-ketamine (Narketan®; 40 mg/kg) and dexmedetomidine (Dexdomitor®; 0.25 mg/kg) and maintained for approximately 30-60 min. Immediately after measurements, atipamezole (1 mg/kg) was injected subcutaneously. To perform aABR recordings animals were placed in a custom-designed sound-attenuating box, and disposable 27-gauge subdermal needle electrodes (Rochester Electro-Medical Inc., Lutz, FL, USA) were placed behind the pinna of the right ear (active) and at the vertex (reference). The ground electrode was placed in the right flank of the animal. The aABR recordings were performed using the procedure described previously by Ramekers et al. [34] with some slight modifications. Broadband acoustic clicks (20 *μ*s monophasic rectangular alternating pulses; interstimulus interval 99 ms; n=75 pairs) were generated and attenuated using an RZ6-A-P1 Auditory Processor (Tucker-Davis Technologies Inc., Alachua, FL, USA). The responses were differentially amplified (×5,000), band-pass filtered (0.1-10 kHz) using a PAR-5113 preamplifier (EG&G Instruments, Gaithersburg, MD, USA), digitized, sampled at 195 kHz, and stored on a personal computer for offline analysis. Stimulus generation and signal acquisition were controlled with custom-made software (Department of Medical Engineering and Clinical Physiology, University Medical Center Utrecht).

Acoustic clicks were delivered monaurally using ER-2 Tubephone™ Insert Earphones (Etymotic Research Inc., Elk Grove Village, IL, USA) and 3.5 mm E-A-RLink Infant Eartips (E-A-R® Auditory Systems, Indianapolis, IN, USA), first to the right ear and next to the left ear. Hearing thresholds were obtained by starting at approximately 100 dB peak-equivalent sound pressure level (SPL) and decreasing the sound level in steps of 10 dB until the response had disappeared. Peak thresholds were then defined as the interpolated sound level at which the aABR peak was 0.3 *μ*V. Only waves I and II were analyzed, since these are more discernable than later peaks. Data were processed using MATLAB software (MathWorks, Natick, MA, USA) and MS Excel (Microsoft, Redmond, WA, USA).

### 2.5. Histology

#### 2.5.1. Fixation and Tissue Processing

Immediately after the final aABR measurements, the animals were euthanized by an intracardial injection with sodium pentobarbital (Euthasol®; AST Farma, Oudewater, the Netherlands). After decapitation, both cochleas were removed and fixed by means of intralabyrinthine perfusion with a fixative containing 3% glutaraldehyde, 2% formaldehyde, 1% acrolein, and 2.5% dimethyl sulfoxide in 0.08 M sodium cacodylate buffer, pH 7.4 [35]. Perfusion was followed by overnight immersion in the same fixative at 4°C, after which the cochleas were rinsed several times in 0.1 M sodium cacodylate buffer (pH 7.4). Cochleas were decalcified in a 10% aqueous EDTA.2Na solution (pH 7.4) at room temperature for 5-7 days and postfixed in a 1% aqueous OsO_4_ solution containing 1% K_4_Ru(CN)_6_ at 4°C for 2 h followed by several rinses in distilled water. Dehydration was performed in a graded ethanol (50-100%) and propylene oxide series. Cochleas were embedded in Spurr's low-viscosity resin which was allowed to polymerize at 70°C overnight. Next, the cochleas were divided into two halves along a standardized midmodiolar plane [36] and reembedded in fresh resin. For histological evaluation, 5 consecutive semithin (1 *μ*m) sections were cut with a diamond knife on a Leica RM2265 microtome, collected on gelatin-coated glass slides, stained with an aqueous solution of 1% methylene blue, 1% azur B, and 1% sodium tetraborate, and mounted in Entellan® (Merck KGaA, Darmstadt, Germany) mounting medium under a glass coverslip.

Sections were examined with a Leica DM5500B microscope equipped with a Leica DFC 450C digital color camera. Digital images of each transection of Rosenthal's canal (two basal, two middle, and three apical transections) as well as the corresponding sections of the organ of Corti were acquired and stored using Leica Application Suite software (LAS V4.5; Leica Microsystems GmbH, Wetzlar, Germany).

#### 2.5.2. Hair Cell Counts

From base to apex the guinea pig cochlea spirals upwards in approximately 4 turns. The number of hair cells (OHCs and IHCs) was counted in a semiquantitative way as described by Van Ruijven et al. [36]. This protocol does not involve the time-consuming preparation of cytocochleograms by counting all (remaining) hair cells within the cochlea but limits itself to 7 different locations along the cochlear spiral at a half-turn spacing, as seen in semithin sections of cochleas divided along a standardized midmodiolar plane (B1, B2, M1, M2, A1, A2, and A3; Figure 1).

Hair cell counts are expressed as the mean percentage of remaining hair cells per individual transection of the respective half-turn (two transections each for the basal [B1, B2] and middle [M1, M2] turns and three transections for the apical [A1, A2, A3] turn).

In oblique transections of the organ of Corti, either one of the following features was used for the identification of OHCs: (1) the presence of the reticular lamina and/or stereocilia; (2) the degree of cytoplasmic staining characteristic for OHCs; and (3) the presence of the basal part of the hair cell. All remaining OHCs were counted irrespective of their histological appearance. OHCs and IHCs were counted at each location using the Cell Counter plugin of ImageJ (https://imagej.nih.gov/ij/plugins/cell-counter.html). Counted hair cells were marked in the images of the histological sections to prevent double counting. For statistical analysis, OHC counts were averaged for each cochlear turn (basal, middle, and apical) within each treatment group and compared to a dataset of averaged OHC counts from the left (nontreated) cochleas.

#### 2.5.3. Spiral Ganglion Cell Packing Densities

SGC packing densities were determined in digital images (acquired at a fixed objective lens magnification of x20) of Rosenthal's canal taken from 5 different cochlear locations (B1, B2, M1, M2, and A1) as previously described by Versnel et al. [37] and Agterberg et al. [38]. Using ImageJ image analysis software (version 1.47; US National Institutes of Health, Bethesda, MD, USA), the bony boundaries of Rosenthal's canal were outlined and its cross-sectional area (in *μ*m^2^) was calculated.

SGC perikarya were counted at each location using the Cell Counter plugin of ImageJ (https://imagej.nih.gov/ij/plugins/cell-counter.html), including (1) all perikarya demonstrating the morphological determinants typical of type I and type II SGCs (no distinction was made between type I and type II SGCs; for details, see Romand and Romand [39]); (2) partial and complete profiles of perikarya; and (3) perikaya with and without evident nucleus or nucleoli. The SGC packing density was calculated by dividing the number of SGCs by the cross-sectional area of Rosenthal's canal and expressed as the mean number of SGCs per mm^2^.

For statistical analysis, SGC packing densities for each cochlear turn (basal, middle, and apical) were averaged within each treatment group and compared to a dataset of averaged SGC packing densities from the left (nontreated) cochleas.

#### 2.5.4. Stria Vascularis

We evaluated in a qualitative manner the following morphological features: shrinkage and/or swelling of the marginal and intermediate cells, edema, loss of intermediate cells (resulting in flattening of the stria vascularis), and the degree of azurophilic staining of the marginal and intermediate cells (intracellular density).

### 2.6. Data Analysis

For both aABR wave I and wave II separately, the thresholds and amplitudes were grouped first by treatment (10 mM, 1 mM, 0.1 mM, 0.01 mM ouabain, and PBS alone) and subsequently by histological appearance. The threshold was defined as the interpolated sound level at which a 0.3 *μ*V signal was evoked. The aABR amplitude before treatment (Day 0) was defined as the largest amplitude recorded for that particular wave; at subsequent time points the amplitude was determined at the same sound level (typically ~90 dB SPL). Changes in threshold and amplitude after ouabain treatment were both expressed in dB relative to pretreatment values.

Statistical analyses of the data were performed using MS Excel and GraphPad Prism 7.00 software (GraphPad Software, La Jolla, CA, USA). Data were expressed as mean ± standard deviation (SD). A one-way ANOVA with Tukey's multiple comparison test and 95% confidence interval was applied to the data. Correlations between aABR measures and OHC counts were assessed with the nonparametric Spearman's rank correlation coefficient; for correlations between aABR measures and SGC packing densities the parametric Pearson's correlation coefficient was used. Groups I-V were compared using ANOVA. Due to small group sizes (i.e., Group I and Group V) it is difficult to check assumptions, and therefore an additional statistical analysis (sensitivity analysis) was performed by pooling groups into three final groups (low treatment dose [L]: 0 mM (i.e., PBS alone), 0.01 mM; medium treatment dose [M]: 0.1 mM; high treatment dose [H]: 1 mM, 10 mM) to achieve minimum group size of four animals. Gender was included in a two-way ANOVA to test for possible gender effects.

## 3. Results

### 3.1. Auditory Brainstem Responses

**Figure 2:**
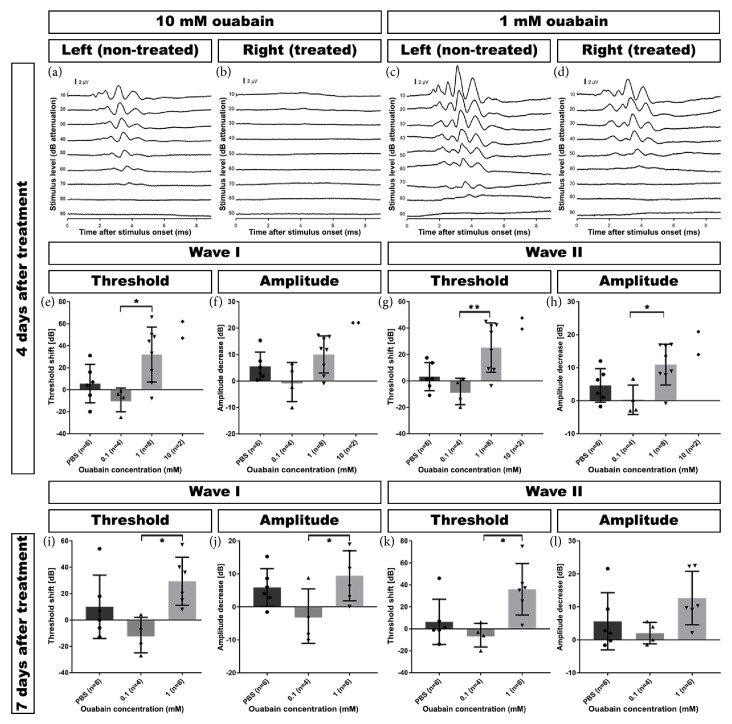
Acoustically evoked auditory brainstem responses (aABRs) were recorded before and after treatment with ouabain. (a+b) Example of an animal treated with 10 mM ouabain revealing a dramatic aABR threshold shift in the right (treated) cochlea (b), while the responses in the left (untreated) cochlea (a) remained normal. (c+d) Example of an animal treated with 1 mM ouabain showing a moderate increase of the aABR threshold in the right (treated) cochlea (d), while the aABR threshold in the left (untreated) cochlea (c) remained normal. (e) Wave I threshold increased substantially after treatment with 1 mM and 10 mM ouabain. There was no significant threshold shift after treatment with lower concentrations (0.1 mM and 0.01 mM). Left (untreated) ears and right ears treated with PBS alone served as control. (f) Four days after treatment, wave I amplitudes had also decreased substantially in the cochleas treated with 1 mM and 10 mM ouabain, but not with lower concentrations (0.1 mM and 0.01 mM). Left (untreated) ears and right ears treated with PBS alone served as control. (g) Within 4 days after treatment with 1 mM and 10 mM ouabain, wave II threshold increased substantially. There was no significant threshold shift after treatment with lower concentrations (0.1 mM and 0.01 mM). Left (untreated) ears and right ears treated with PBS alone served as control. (h) Wave II amplitudes also decreased substantially in the cochleas treated with 1 mM and 10 mM ouabain 4 days after treatment, but not with lower concentrations (0.1 mM and 0.01 mM). Left (untreated) ears and right ears treated with PBS alone served as control. (i) Seven days after treatment, wave I thresholds were still substantially elevated in animals treated with 1 mM ouabain. There was no significant threshold shift after treatment with lower concentrations (0.1 mM and 0.01 mM). Left (untreated) ears and right ears treated with PBS alone served as control. (j) Wave I amplitudes remained substantially decreased in cochleas 7 days after treatment with 1 mM ouabain. Animals treated with lower concentrations (0.1 mM and 0.01 mM) did not display significant decrease in wave I amplitude. Left (untreated) ears and right ears treated with PBS alone served as control. (k) Wave II thresholds remained substantially elevated in animals 7 days after treatment with 1 mM ouabain. There was no significant threshold shift after treatment with lower concentrations (0.1 mM and 0.01 mM). Left (untreated) ears and right ears treated with PBS alone served as control. (l) Seven days after treatment with 1 mM ouabain, wave II amplitudes remained substantially decreased in these animals. Animals treated with lower concentrations (0.1 mM and 0.01 mM) did not display significant decrease in wave II amplitude. Left (untreated) ears and right ears treated with PBS alone served as control. Thresholds (e, g, i, and k) and amplitudes (f, h, j, and l) are expressed as change (in dB) from pretreatment levels. Differences in amplitudes of contralateral ears between animals treated with 1 mM and 10 mM are within the normal range of the animals. Error bars represent SD; *∗* = p ≤ 0.05, *∗∗* = p ≤ 0.01, *∗∗∗* = p ≤ 0.001.

All animals showed normal-hearing thresholds prior to surgery (Day 0) without significant differences among the treatment groups (wave I: p = 0.52; wave II: p = 0.25; one-way ANOVA, Tukey's multiple comparison test, 95% confidence interval). This was evaluated by comparison to normative data from previous studies from our group [40, 41]. In addition, no significant differences were found between waves I and wave II within the different treatment groups at the different days (Days 0, 2, 4, and 7). Since wave II was more robust and less prone to random fluctuation than wave I in our measurements, we predominantly focus on the wave II data as described previously [42]. Figures 2(a)–2(d) depict representative aABRs of the left (nontreated) and right (treated) ears, 4 days after treatment with ouabain.

After treatment with 10 mM ouabain (Group I) the right ears showed a substantial and statistically significant 43-dB increase in the wave II thresholds, already within 4 days (p < 0.005; one-way ANOVA; Figure 2(g)), whereas the left (nontreated) ears, as expected, did not show any threshold shift. The left ear showed a normal aABR (Figure 2(a)), while the right ear revealed substantial hearing loss that resulted in one-sided deafness after treatment with 10 mM ouabain (Figure 2(b)).

After 1 mM ouabain, the left (nontreated) ear was not affected (Figure 2(c)), but the right (treated) ear exhibited a hearing loss of 30 dB (Figure 2(d)). Of the 8 animals treated with 1 mM ouabain (Group II), 2 animals did not show any changes in their aABR thresholds of both wave I and wave II (i.e., nonresponders). The remaining 6 animals expressed pronounced changes in wave II thresholds. On average, the 8 animals in Group II demonstrated an increase in wave II threshold of the right ears, already within 4 days (25 dB; p = 0.585; one-way ANOVA; Figure 2(g)), and did not change at Day 7 (24 dB). The left (nontreated) ears showed normal-hearing thresholds.

The decreases in wave II amplitudes were significantly different in Groups I and II (p = 0.007 and p = 0.015, respectively; one-way ANOVA), 4 days after deafening (Figure 2(h)). The pattern of amplitude decrease across treatment groups resembles the increase in threshold as shown in Figure 2(g).

Animals treated with lower concentrations of ouabain, i.e., 0.1 mM (Group III) and 0.01 mM (Group IV) or PBS alone (Group V), did not show any significant changes in the threshold and amplitude of wave II. The left (nontreated) ears also showed normal-hearing thresholds. aABR latencies were unaffected in any of the groups at any time point after ouabain treatment (data not shown).

Including gender in the ANOVA did not change the results (e.g., p = 0.7 for the model amplitude of wave II at Day 4 against treatment Groups I-V). The p values for gender were larger than 0.7 in all analyses. Concerning the additional sensitivity analysis, using the pooled groups (L and H) also did not change the results in most analyses (e.g., p = 0.0058 no pooling [I-V], p = 0.0047 pooled [L, M, and H] treatment groups, amplitude of wave II at Day 4 against treatment groups). For analyses of wave I, the significance of the sensitivity analysis for the pooled treatment groups increased as compared to the original group allocation (e.g., p = 0.02 no pooling [I-V], p = 0.0056 pooled [L, M, and H] treatment groups, amplitude of wave I at Day 4 against treatment groups). This can be explained by the fact that the effects for low treatment concentrations are similar and pooled into the same pooled group. In all cases p values for the sensitivity analysis of the pooled treatment groups were lower than for the original group allocation. This implies that the analyses presented above are robust with respect to group size.

### 3.2. Histology

#### 3.2.1. General Findings

**Figure 3:**
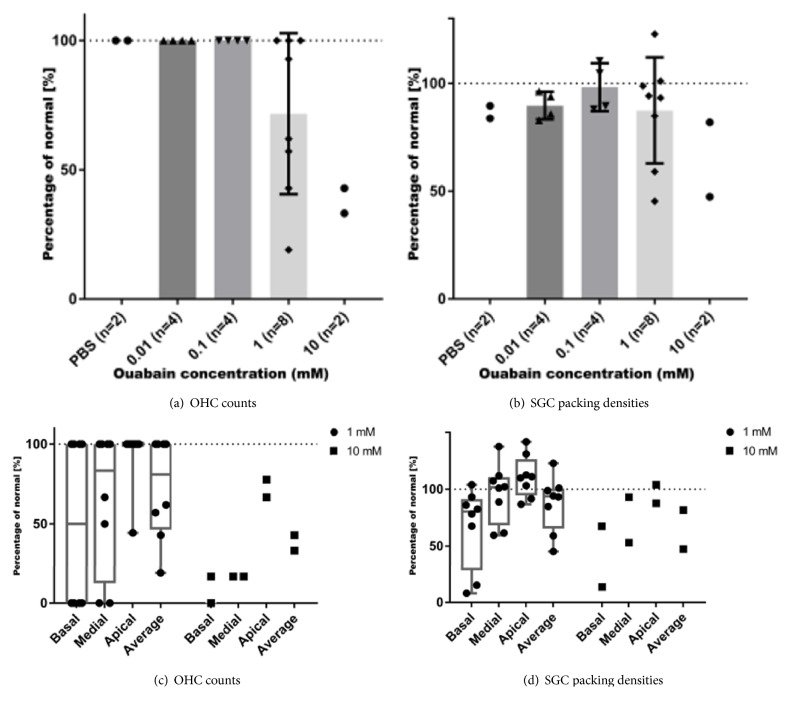
Quantification of OHC loss and SGC loss after ouabain treatment. Data are normalized and expressed as percentages of an averaged dataset from the left (untreated) cochleas (normal). (a) OHC loss accrues with increasing ouabain concentrations (1 mM: 72.6 ± 11.0% of OHCs remaining; 10 mM: 38.1 ± 4.8% of OHCs remaining). IHCs are not affected. Control (0 mM) includes right ears treated with PBS alone. Normal (100%) is 21 OHCs, the sum of OHCs counted at B1, B2, M1, M2, A1, A2, and A3. (b) SGC loss also progresses with increased ouabain concentrations. Compared to SGC packing densities in the left (nontreated) cochleas, near-normal numbers (95.3 ± 2.8%) of the SGC perikarya are present in cochleas treated with 0.01 mM and 0.1 mM ouabain. Control (0 mM) includes right ears treated with PBS alone. Normal (100%) is 1362 SGCs/mm^2^, the average SGC packing density determined in B1, B2, M1, M2, and A1. Cochleas treated with 1 mM ouabain show a moderate loss with an average of 87.4 ± 8.7% of SGC perikarya remaining, whereas in cochleas treated with 10 mM ouabain the average number of SGC perikarya present in Rosenthal's canal decreases to 64.6 ± 17.2%. Error bars represent SD; *∗∗* = p ≤ 0.01, *∗∗∗* = p ≤ 0.001. (c) OHC counts in the basal, middle, and apical turns at 1 mM and 10 mM ouabain and the average OHC count (cf. Figure 3(a)). For basal and middle turns, normal (100%) is 6 OHCs per turn, i.e., the sum of OHCs counted at B1+B2 and M1+M2, respectively, and for the apical turn normal (100%) is 9 OHCs, the sum of OHCs counted at A1+A2+A3. At a concentration of 1 mM, ouabain causes OHC loss in the basal (50.0 ± 18.9%) and middle (35.4 ± 15.6%) turns. In the apical turn, OHC counts are almost normal (93.1 ± 7.0%). A concentration of 10 mM ouabain results in loss of OHCs in all turns. In the basal turn 91.7 ± 8.4% and in the middle turn 83.3% of the OHCs are lost. In the apical turn, approximately 27.8 ± 5.5% of the OHCs is lost. (d) SGC packing densities in the basal, middle, and apical turns at 1 mM and 10 mM ouabain and the average SGC packing density (cf. Figure 3(b)). Normal (100%) is 1293 SGCs for the basal turns, 1470 SGCs for the middle turns, and 1325 SGCs for the apical turn. In the basal turn, SGC packing densities are reduced to 66.9 ± 12.6%, while they are near-normal in the middle (96.2 ± 9.2%) and apical (111.0 ± 6.5%) turns. At a concentration of 10 mM ouabain, SGC packing densities are reduced to 40.5 ± 17.0% in the basal turn and 73.1 ± 20.1% in the middle turn. The packing density in the apical turn is normal (95.8 ± 8.1%).

Table 1 summarizes the general histological findings. We found OHC loss and SGC loss in animals from Group I and in some of Group II, but none of the animals demonstrated loss of IHCs or morphological changes in the stria vascularis, such as swelling, shrinkage, edema, or gross changes in intracellular density. In the right cochleas of both animals in Group I (10 mM ouabain) a near-complete loss of OHCs was seen in the basal and middle turns and in the lower region of the apical turn, either with (n=1) or without (n=1) SGC loss. The average numbers of OHCs present in all turns of the cochlea amounted to 38.1 ± 4.8% (p < 0.001, one-way ANOVA; Figure 3(a)) and 64.6 ± 17.2% of the SGCs were present on average (Figure 3(b)).

**Figure 4:**
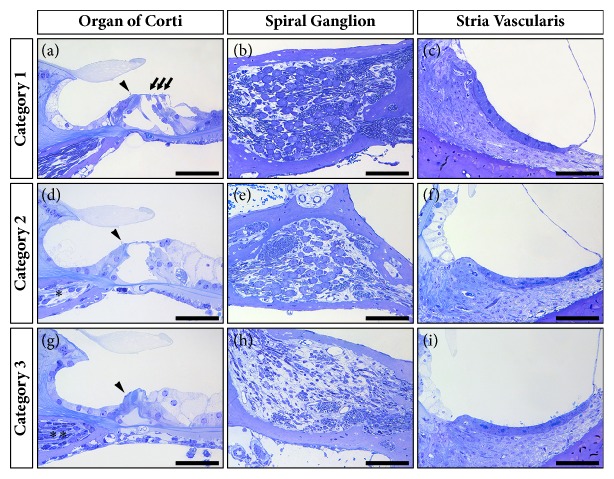
Examples of the variety in histological changes in the cochlea after round window membrane application of ouabain in guinea pigs. Cochleas in Category 1 do not demonstrate any effect of ouabain upon the OHCs (arrows), IHCs (arrow heads), SGCs, or the stria vascularis. These cochleas do not appear different from the left (nontreated) cochleas or from the right cochleas treated with lower ouabain concentrations (0.1 mM and 0.01 mM) or PBS alone. Category 2 contains cochleas with complete OHC loss, with apparent loss of peripheral processes in the osseous spiral lamina (*∗*), but without obvious loss of SGCs, in the basal and middle turns, while cochleas in Category 3 show complete OHC loss in all turns together with loss of peripheral processes in the osseous spiral lamina, which seem to be substituted with fibroblasts (*∗∗*), and extensive loss of SGCs in the basal and middle turns. In none of the animals, any loss of IHCs or histological changes in the stria vascularis are obvious. Scale bars: 50 *μ*m (organ of Corti) and 100 *μ*m (spiral ganglion and stria vascularis).

Of the 8 animals treated with 1 mM ouabain (Group II), 2 animals did not show any changes in their aABR thresholds. In addition, these nonresponding animals did not show any morphological changes in the right (treated) cochleas. The remaining 6 animals demonstrated a significant increase of their wave II thresholds as well as varying degrees of histological changes in their right cochleas. Two animals did not show any histological changes, despite the observed threshold shift in wave II. Another 2 animals demonstrated complete OHC loss (but no loss of IHCs) in the basal (B1, B2) and middle (M1, M2) turns of the right cochleas. Although there was no obvious loss of SGC perikarya, the number of peripheral processes in the osseous spiral lamina appears diminished (see Figure 4(d)) or the peripheral processes seem to have been substituted by fibroblasts (Figure 4(g)). In the remaining 2 animals, OHC loss was seen in all turns of the right cochleas (complete loss in the basal (B1, B2) and middle (M1, M2) turns and partial loss in the apical (A1, A2) turn) together with loss of peripheral processes in the osseous spiral lamina as well as a complete loss of SGC perikarya (type I and type II) in the basal (B1, B2) turn and partial loss in the lower middle (M1) turn.

Statistical analysis of the averaged OHC counts and SGC packing densities demonstrates that the cochleas in Group II exhibit a significant OHC loss (28.3 ± 11.0%; p = 0.004, one-way ANOVA; Figure 3(a)) together with minor loss of SGCs (12.6 ± 8.7%; Figure 3(b)).

Animals in Groups III to V did not show any obvious morphological changes 7 days after treatment with ouabain or decreased OHC counts or SGC packing densities (Figures 3(a)-3(b)). The left (nontreated) cochleas all demonstrated a normal morphology.

To investigate the longitudinal (basal-apical) gradient of the cochleotoxic effect of ouabain, we analyzed the OHC counts of animals treated with 1 mM and 10 mM ouabain from the basal, middle, and apical turns. At 1 mM (Group II), ouabain caused OHC loss in the basal (50.0 ± 18.9%) and middle (35.4 ± 15.6%) turns, whereas in the apical turn OHC counts were close to normal (93.1 ± 7.0%; Figure 3(c)). At a concentration of 10 mM ouabain OHC loss was seen in all turns: in the basal turn 91.7 ± 8.4% of the OHCs were lost, in the middle turn 83.3% of the OHCs were lost, and in the apical turn 27.8 ± 5.5% of the OHCs were lost (Figure 3(c)).

SGC packing densities were reduced primarily in the basal turn (66.9 ± 12.6%) but were near-normal in the middle and apical turns of guinea pigs treated with 1 mM ouabain (Figure 3(d)). At a concentration of 10 mM ouabain, SGC packing densities were reduced to 40.5 ± 17.0% in the basal turn and 73.1 ± 20.1% in the middle turn. In contrast to the OHC counts at 10 mM ouabain, the SGC packing density in the apical turn remained normal (95.8 ± 8.1%).

#### 3.2.2. Categorization Based upon Histological Findings

Variability within Groups I and II was substantial, and the observed loss of both OHCs and SGCs was often bimodal; whereas some animals showed substantial losses, others in the same group did not. Therefore, we pooled all histological data of the right cochleas in all groups and defined three categories based upon the general histological observations (Figure 4; Table 2):* Category 1* (n=14) contains cochleas that do not demonstrate any loss of OHCs (Figure 4(a)) and SGCs (Figure 4(b)); in* Category 2* (n=3) the cochleas show OHC loss only (Figures 4(d)-4(e)); and in* Category 3 *(n=3) the cochleas exhibit both OHC and SGC loss (Figures 4(g)-4(h)). OHC loss was defined as the absence of any of the 3 OHCs in any of the 7 transections of the organ of Corti. SGC loss was defined as a loss of ≥20% as compared to the averaged SGC packing densities of the left (nontreated) cochleas (n=12). In none of the animals IHC loss (Figures 4(a), 4(d), and 4(g)) or any morphological changes in the stria vascularis (Figures 4(c), 4(f), and 4(i)) were obvious.

**Figure 5:**
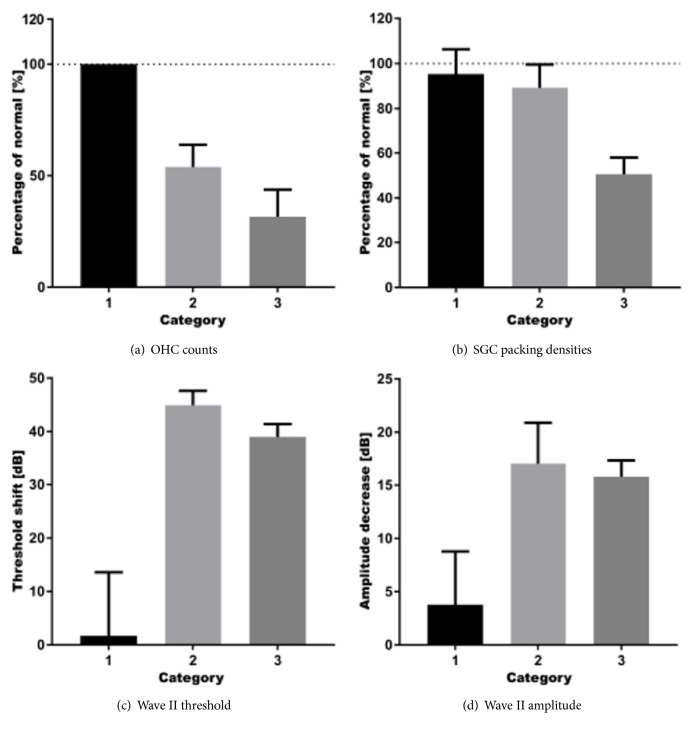
Reevaluation of results using* post hoc* histological categorization. (a) Category 1 consists of cochleas without any OHC loss. In Category 2, average OHC loss is moderate and in Category 3 the average number of remaining OHC has even decreased more. Normal (100%) is 21 OHCs, the sum of OHCs counted at B1, B2, M1, M2, A1, A2, and A3. (b) Average SGC packing density in Category 1 cochleas is near-normal. There is on average minor loss of SGCs (i.e., <20%) in Category 2, whereas the average SGC packing density in Category 3 cochleas is approximately half of that in normal-hearing controls, i.e., the left (nontreated) cochleas. Normal (100%) is 1362 SGCs/mm^2^, the average SGC packing density determined in B1, B2, M1, M2, and A1. (c) The wave II threshold of Category 1 remains normal, while animals in Categories 2 and 3 showed a substantial shift in wave II threshold, 4 days after treatment with ouabain. The wave II threshold shifts do not differ between Categories 2 and 3. (d) Animals in Category 1 show a slight decrease in their wave II amplitude, whereas animals in both Categories 2 and 3 show a much larger decrease, 4 days after ouabain treatment. The wave II amplitudes in Categories 2 and 3 do not significantly differ from one another. OHC counts (a) and SGC packing densities (b) are normalized and expressed as percentages of an averaged dataset from the left (untreated) cochleas (normal). Thresholds (c) and amplitudes (d) are normalized and expressed as change (in dB) from pretreatment levels. Error bars represent SEM.

Reevaluation based upon the averaged OHC counts and SGC packing densities shows that cochleas in Category 1 exhibit normal numbers of OHCs (100%) and SGCs (95.3 ± 3.0%), comparable to the left (nontreated) cochleas, together with near-normal wave II amplitudes and normal-hearing thresholds (Figure 5).

Cochleas of Category 2 showed considerable OHC loss (46.0 ± 5.7%; Figure 5(a)) and on average minor loss of SGCs (10.8 ± 6.0%; Figure 5(b)), while the animals displayed substantial threshold shifts and substantially decreased wave II amplitudes as compared to those in Category 1 (Figures 5(c)-5(d)).

In the cochleas of Category 3 a substantial number of the OHCs (68.3 ± 6.9%) was lost, as compared to Categories 1 and 2 (Figure 5(a)), and SGC loss was considerably higher (49.4 ± 4.3%) than that in Categories 1 and 2 (Figure 5(b)). Animals in Category 3 showed a substantial decrease in wave II amplitudes as well as a substantial threshold shift, similar to those in Category 2 (Figures 5(c)-5(d)).

**Figure 6:**
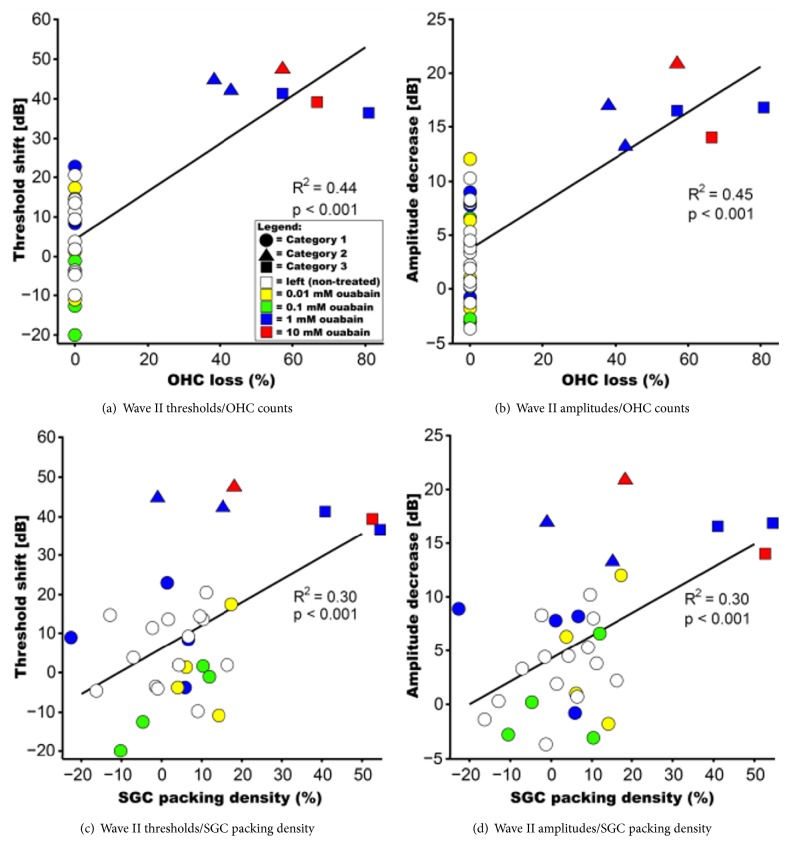
Scatter plots of aABR data and OHC loss and SGC packing densities. OHC counts and SGC packing densities are normalized and expressed as percentages of an averaged dataset from the left (untreated) cochleas. Thresholds and amplitudes are normalized and expressed as change (in dB) from pretreatment levels. (a) The variance in wave II threshold shift after ouabain treatment can be explained for 44% by OHC loss (p < 0.001). (b) The decrease in wave II amplitude is positively correlated with OHC loss. The explained variance (R^2^) is 45% (p < 0.001). (c) The aABR wave II threshold shift and SGC loss are moderately correlated (p < 0.001). (d) There is also a moderate positive correlation between wave II amplitude decrease and SGC packing densities (p < 0.001). Colors indicate treatment group: open symbols are left (nontreated) cochleas; right ears are shown in closed symbols colored yellow (0.01 mM ouabain), green (0.1 mM), blue (1 mM), and red (10 mM).* Post hoc* categorization is visualized using circles (Category 1), triangles (Category 2), and squares (Category 3).

Although the right cochleas of animals in Category 2 had, on average, near-normal SGC packing densities, their ABR thresholds had increased and their ABR amplitudes had decreased. It seems that, in this particular category, OHC loss is a more sensitive measure to determine hearing loss. Therefore, we investigated how the quantitative histological data correlate with the aABR data (Figure 6). The results show a moderately positive correlation between the variance in threshold shift after ouabain treatment and OHC loss (Figure 6(a); R^2^ = 0.44, p < 0.001); the decrease in wave II amplitude correlated to a similar extent with OHC loss (Figure 6(b); R^2^ = 0.45, p < 0.001). However, in both cases the relationship between the two variables clearly deviated from a linear one; these data should therefore be treated with caution. The positive correlations between threshold shift and SGC loss and between amplitude decrease and SGC loss (Figures 6(c)-6(d); for both R^2^ = 0.30; p < 0.001) is weak and indicates that the number of SGCs is not primarily related to audiometric threshold shifts. We confirmed that, throughout all experiments, correlations between aABR wave II measurements (threshold/amplitude) and histological quantifications (OHC/SGC counts) were highly similar to those for wave I (data not shown).

## 4. Discussion

We found that, within days, locally applied ouabain, in a dose-dependent fashion, results in a loss of OHCs, either with or without concomitant SGC loss. Both OHC loss and SGC loss demonstrated a longitudinal gradient with a preference for the basal and middle turns, similar to the effect of various other cochleotoxic drugs. Loss of IHCs and morphological changes in the stria vascularis were not seen. These results are largely in line with the observations of Hamada and Kimura [32], who reported that round window application of ouabain results in morphological damage to three main targets in the guinea pig cochlea, i.e., the organ of Corti, the spiral ganglion, and the stria vascularis. Similar to our results, degeneration of OHCs was one of their most consistent findings. In addition, they reported that IHCs often remained intact after ouabain treatment and that in some cochleas severe shrinkage of type I SGCs was present; there are even indications of loss of SGC perikarya (their Figure  1E).

The stria vascularis in our samples did not demonstrate any gross morphological changes, such as cell swelling or shrinkage of the marginal and intermediate cells, as previously reported after perilymphatic perfusion of 2 mM ouabain [43]. Nor did we observe an increase in the intercellular spaces (edema) as seen by Hamada and Kimura [32] after round window application of 2 mM ouabain, which they describe as ‘cystic stria vascularis' (their Figure  1D). It should be noted that they only found this strial edema in the most severely affected cases. Neither did we observe intracellular vacuolation of the marginal cells as reported in guinea pigs after cochlear perfusion of 1 mM ouabain using a glass perfusion pipette [44].

After round window membrane application, ouabain directly enters into the perilymph of the scala tympani. The observed ouabain-induced degeneration of OHCs can be explained by one or more of the following mechanisms.

Ouabain could exert direct effect upon the Na^+^/K^+^-ATPase present in the basolateral membranes of the OHCs. Their main body is immersed in cortilymph, which communicates with the scala tympani. Perilymphatic ouabain may diffuse into the cortilymph [45] and subsequently bind to the Na^+^/K^+^-ATPase in the basolateral membranes of the OHCs, resulting in either inhibition of its ion transporter function or disturbance of its signal receptor function [46]. Ouabain-induced degeneration of OHCs may therefore be attributed to a direct effect upon these cells, although, alternatively, OHC loss may be the result of a direct effect upon the stria vascularis resulting in changed electrolyte composition of the endolymph and, hence, of the endocochlear potential [43, 47, 48].

It has been shown that perilymphatic perfusion of ouabain interferes with strial function resulting in a changed electrolyte composition of the endolymph and reduced cochlear potentials [43, 44]. It has generally been assumed that this effect is based upon direct binding of ouabain to the Na^+^/K^+^-ATPase located at the basolateral membranes of the marginal cells, which are involved in secretion of K^+^ into the endolymph and thus maintain endolymph homeostasis and generation of the endocochlear potential. For this to take place, it is necessary that perilymphatic ouabain enters the stria vascularis after diffusion through the loosely arranged collagen network of the spiral ligament (cf. [45, 49]), although there is no experimental evidence for such a diffusion route. A recent paper by Adachi et al. [50] concludes that fibrocytes in the spiral ligament play a pivotal role in the transport of K^+^ to the stria vascularis and, hence, are involved in the maintenance of the endocochlear potential. From their data it seems likely to propose that ouabain exerts a direct effect upon the Na^+^/K^+^-ATPase present in the type II spiral ligament fibrocytes rather than upon the strial Na^+^/K^+^-ATPase.

The subsequent change in endolymph composition could then (indirectly) impair the function of the OHCs. Although no visible morphological changes in the stria vascularis (and the spiral ligament) could be observed in our animals, it cannot be excluded that ouabain has interfered with both tissues at a subcellular level.

We observed that treatment with ouabain resulted in complete loss of the OHCs, primarily in the basal and middle turns, already within 4-7 days after round window membrane application of ouabain. This rapid loss of OHCs is similar to that observed after concomitant administration of kanamycin and furosemide [37], which results in a complete loss of OHCs in all cochlear turns within 7 days.

Loss of IHCs was not observed in any of the responding animals. This is in line with a myriad of earlier publications, indicating that IHCs are less vulnerable to most ototoxic drugs. Although we did not observe any morphological changes at the light microscopical level, it cannot be excluded that cellular changes may have occurred in the IHCs, as illustrated by the observation that ouabain-induced loss of SGCs eventually results in loss of the synaptic ribbons in the IHC [20]. This so-called hidden hearing loss has been recently demonstrated to occur in noise-exposed or aged cochleas in mouse models [51].

The effect of ouabain upon the SGCs may be taking place parallel to that upon the OHCs, but loss of SGCs seems to be a later event, as illustrated by our observation that SGCs are still present in a number of animals in which complete OHC loss had occurred. On the other hand, we observed that in some animals loss of OHCs was accompanied by SGC loss. This implies that ouabain may act simultaneously upon the perikarya of the SGCs and/or the surrounding satellite glial cells or Schwann cells, which has also been reported for cisplatin-induced cochleotoxicity [36]. Ouabain can directly affect the Na^+^/K^+^-ATPase present in the nerve fibres, the perikarya of the SGCs, and/or the satellite glial cells leading to depolarization of the cell and eventually neuronal cell death [52], because ouabain can enter Rosenthal's canal through the canaliculi perforantes which are located in the osseous spiral lamina and the lateral wall of the bony modiolus [45, 53]. The observed effect of ouabain upon the SGCs is more acute than that after combined administration of kanamycin and furosemide. With ouabain concentrations of ≥1 mM, already within 7 days a complete loss of SGCs can be observed, whereas with the kanamycin and furosemide deafening protocol it takes 4-6 weeks to obtain such a dramatic loss [37, 54].

Our data clearly show that ouabain interferes with auditory function in guinea pigs, as both the aABR amplitude and threshold were affected, seemingly in a dose-dependent fashion, already within 2 days after round window membrane application, and did not recover during the course of the experiment (cf. Figure 2). However, since the histological damage varied considerably within these treatment groups, it is difficult to identify the main cause for the observed hearing loss. The high variability in histological damage observed in the (affected) animals (Groups I and II) may be due to the fact that gelfoam was used as a carrier to apply ouabain to the cochlea. It is not certain if with this means of application ouabain concentrations have reached equal intracochlear levels among the different animals. However, especially the results of Group I (10 mM) have to be handled with caution, since this group only consists of two animals and thus displays results of a group with limited sample size. The small number of animals causes high uncertainty about the variability in this group. Some variability in our method might also be the result of outbred albino Dunkin Hartley guinea pigs, which are commonly used in inner ear biology. One method to possibly reduce this variability is to use inbred strains, such as strain 2 or strain 13, for future experiments [55]. Our sensitivity analyses suggest that the groups treated with PBS and 0.01 mM ouabain are similar as well as the groups treated with 1 mM and 10 mM ouabain as pooling these pairs of groups did not cause a dilution of effect in either the ANOVA or the linear regression. Nonetheless, the histological results of Group I (10 mM) and Group II (1 mM) suggest that ouabain, when used in the guinea pig to induce selective degeneration of (type I) SGCs, is contrary to the observations in other rodent species since it also results in loss of OHCs.

The* post hoc* categorization of the data based upon our histological observations reveals that cochleas exhibiting only moderate OHC loss without any significant SGC loss (Category 2) displayed a degree of hearing loss that is rather similar to that observed in cochleas with both severe OHC and SGC loss (i.e., Category 3; see Figure 5). In both categories the threshold shift is substantial and amounts to 39-45 dB, which is in accordance with the 40-45 dB increase in threshold known to be associated with complete OHC loss [56]. The fact that approximately 50% of the OHCs were present in Category 2 and 30% of the OHCs still remained in Category 3 and the remaining OHCs did not show any light microscopical changes may indicate that the function of these remaining OHCs was impaired or that there were just not enough functional OHCs left to perform effective tuning of the basilar membrane. The substantial SGC loss observed in cochleas of Category 3 is not likely to have contributed to the threshold increase, as it would have augmented the difference between the threshold shifts in Category 2 and Category 3 cochleas. Furthermore, thresholds of (electrically evoked) ABRs and compound action potentials do not tend to increase with progressing SGC loss after deafening [34, 42], indicating that a degenerating SGC population in itself does not imply higher excitation thresholds.

The aABR amplitude was substantially lower in the two categories exhibiting OHC loss (Categories 2 and 3; see Figure 5). As with the aABR threshold, the amplitude obviously collapsed with moderate OHC loss but did hardly change with increasing OHC loss and additional SGC loss. Since the aABR amplitude essentially reflects population size, recruitment, and synchrony—and given that the IHCs remained intact—we can therefore conclude that such a substantial decrease in amplitude is likely the result of reduced recruitment caused by OHC loss. It is at this point worthwhile to repeat that our findings were very similar for the ABR wave I and wave II. Despite the histological differences between Categories 2 and 3, the similarity in aABR amplitude and threshold are striking. This discrepancy may partly be explained by histologically intact, but nonfunctional, cells that therefore do not contribute to the mechanoelectric transduction process. With up to 7 days, the time frame of our study was similar to that of the deafening procedure of guinea pigs described by Cho et al. [11]. They found that application of ouabain results in severe decrease in SGC numbers within 7 days. In our study, this histological effect can be observed within the same time frame, including loss of OHCs. However, we only studied the effect of ouabain on OHCs, SGCs, and stria vascularis over a time frame of up to 7 days. Consequently, we can only draw conclusions about the short-term effect of ouabain upon the OHCs and SGCs, but not about any long-term effects on, for instance, the stria vascularis. In addition, it may be possible that animals treated with 0.1 mM ouabain (Group III) after an extended period, i.e., 14 days or 21 days, do show histological effect. However, regarding the loss of SGCs, a previous study showed that there is no long-term SGC regeneration after systemic deafening [57]. From our experiments we can conclude that ouabain does result in a rapid loss of SGCs in the guinea pig cochlea; in the responding animals already within 7 days a complete loss of SGCs can be observed.

Therefore, in theory, ouabain deafening should be suitable for stem cell therapy in the inner ear since supporting cells and IHCs are preserved. However, the observation that affected aABRs do not automatically implicate an underlying loss of SGCs makes us conclude that locally applied ouabain is not a reliable model to investigate cell-based auditory nerve therapy in the guinea pig.

## Figures and Tables

**Table 1:** Overview of the five experimental groups.

**Group**	**Treatment**	**n**	**ABR Threshold**	**IHC**	**OHC**	**SGC**
1	10 mM ouabain	2	elevated	normal	loss (n=2)	no loss (n=1)
						loss (n=1)

2	1 mM ouabain	8	normal (n=2)	normal	no loss (n=2)	no loss (n=2)
			elevated (n=6)	normal	no loss (n=2)	no loss (n=2)
					loss (n=4)	no loss (n=2)
						loss (n=2)

3	0.1 mM ouabain	4	normal	normal	normal	normal

4	0.01 mM ouabain	4	normal	normal	normal	normal

5	PBS	2	normal	normal	normal	normal

**Table 2:** Distribution of the cochleas from the 5 experimental groups among the three histological categories and corresponding averages.

**Category 1**	**N1**	**Category 2**	**N2**	**Category 3**	**N3**
No OHC and SGC loss		OHC loss only		Both OHC and SGC loss	
Group II	4	Group I	1	Group I	1

Group III	4	Group II	2	Group II	2

Group IV	4				

Group V	2				

Total	14		3		3

OHC loss	0%		46.0 ± 5.7%		68.3 ± 6.9%

SGC loss	4.7 ± 3.0%		10.8 ± 6.0%		49.4 ± 4.3%

Threshold shift	1.7 ± 11.9 dB		44.9 ± 2.7 dB		39.0 ± 2.4 dB

Amplitude	3.8 ± 5.0 dB		17.1±3.8 dB		15.8 ± 1.6 dB

## Data Availability

The authors confirm that the data supporting the findings of this study are available within the article.
